# *Bacillus anthracis* lethal toxin negatively modulates ILC3 function through perturbation of IL-23-mediated MAPK signaling

**DOI:** 10.1371/journal.ppat.1006690

**Published:** 2017-10-23

**Authors:** Sudarshan Seshadri, David S. J. Allan, James R. Carlyle, Lauren A. Zenewicz

**Affiliations:** 1 Department of Microbiology and Immunology, University of Oklahoma Health Sciences Center, Oklahoma City, Oklahoma, United States of America; 2 Department of Immunology, University of Toronto, Toronto, Ontario, Canada; 3 Sunnybrook Research Institute, Toronto, Ontario, Canada; University of Illinois, UNITED STATES

## Abstract

*Bacillus anthracis*, the causative agent of anthrax, secretes lethal toxin that down-regulates immune functions. Translocation of *B*. *anthracis* across mucosal epithelia is key for its dissemination and pathogenesis. Group 3 innate lymphocytes (ILC3s) are important in mucosal barrier maintenance due to their expression of the cytokine IL-22, a critical regulator of tissue responses and repair during homeostasis and inflammation. We found that *B*. *anthracis* lethal toxin perturbed ILC3 function *in vitro* and *in vivo*, revealing an unknown IL-23-mediated MAPK signaling pathway. Lethal toxin had no effects on the canonical STAT3-mediated IL-23 signaling pathway. Rather lethal toxin triggered the loss of several MAP2K kinases, which correlated with reduced activation of downstream ERK1/2 and p38, respectively. Inhibition studies showed the importance of MAPK signaling in IL-23-mediated production of IL-22. Our finding that MAPK signaling is required for optimal IL-22 production in ILC3s may lead to new approaches for targeting IL-22 biology.

## Author summary

*Bacillus anthracis*, the bacterium that causes the deadly disease anthrax, is commonly known for its use in bioterrorism. We have used *B*. *anthracis* to study the effect of MAPK signaling pathways in group 3 innate immune lymphocytes (ILC3s). *B*. *anthracis* enters the host through the lungs or gastrointestinal (GI) tract. To cause disease, the pathogen requires translocation across barrier tissue layers to disseminate. During this infection process, the bacterium produces many virulence factors, including a lethal toxin, that allow it to subvert the host immune response. In many immune cells, lethal toxin turns off signaling events through degradation of intermediate signaling components. In this study, we show that lethal toxin interferes in a specific signaling pathway, MAPK, which is significant because it is not usually associated with IL-23-mediated signaling. We specifically studied the interference of MAPK in ILC3s, rare immune cells found in the lungs and GI tract. These cells secrete high levels of interleukin-22 (IL-22), a key cytokine for maintaining barrier tissues during inflammation. Lethal toxin significantly reduced *in vitro* IL-23-mediated IL-22 production in mouse and human ILC3s. Furthermore, *in vivo* administration of lethal toxin reduced IL-22 production in ILC3s. This negative effect on IL-22 implicated MAPK signaling as one of the required pathways in ILC3s for IL-22 production, which means that *B*. *anthracis* may inhibit ILC3 function during infection, potentially aiding its bacterial dissemination. These findings increase our understanding of the molecular factors that control IL-22 regulation in ILC3s and aid in development of immune therapeutics and preventive measures.

## Introduction

*Bacillus anthracis* is a Gram-positive bacterium that is the causative agent of anthrax, a rare and deadly disease that can infect the host by pulmonary, gastrointestinal (GI) or cutaneous routes [1, 2]. In each route of infection, spores or vegetative bacteria must translocate through an epithelial barrier in order to disseminate and cause disease. Pathogenic bacteria achieve epithelial translocation by secretion of virulence factors that allow the bacteria to physically disrupt barriers, enter host cells, displace commensal bacteria and/or suppress immune responses, allowing the pathogens to disseminate. The mechanisms of bacterial epithelial translocation often involve modulation of host signaling pathways [3, 4]. *B*. *anthracis* secretes lethal toxin, which contributes to barrier disruption via interference of epithelial, endothelial and immune cell function [5].

At the interface between the host and the environment, mucosal barriers have several lines of defense against invasive pathogens such as *B*. *anthracis* [6]. Mucin-rich surfaces and antimicrobial peptides prevent commensal and/or pathogenic bacteria from interacting with the epithelium. Recent studies have revealed a critical role for innate lymphocytes (ILCs) in barrier maintenance [7, 8]. Examining the roles of ILCs in response to commensal and pathogenic bacteria is an area of active investigation. Understanding how pathogenic bacteria may modulate ILC function during infection is a new area of exploration.

ILCs are a broad class of lymphocytes that lack diverse, rearranged antigen-specific receptors [9, 10]. As such, ILCs respond to environmental signals, including cytokines, Toll-like receptor (TLR) ligands and other pathogen-associated molecular patterns (PAMPs). Group 3 innate lymphocytes (ILC3s) are rare immune cells found primarily found in mucosal tissues [9]. ILC3s function in concert with T cells in maintaining tissue homeostasis and protecting the host during bacterial infection [11].

Environmental signals are central for ILC3 activation. The most potent activator of ILC3s is the cytokine IL-23 [12], which is primarily secreted by activated macrophages and DCs. Other signals important for ILC3 activation include IL-1β, TLR ligands and neurotropic factors [13–15]. Activated ILC3s may produce the cytokines IL-22, GM-CSF and to a certain extent IL-17 [10].

IL-22 is one of the most biologically important effector cytokines produced by ILC3s. ILC3s and CD4 T cells are major sources of IL-22 [16]. IL-22 functions as a pro-inflammatory or protective cytokine depending on the context of inflammation [17]. IL-22 is upregulated in many Gram-positive and Gram-negative bacterial infections, including pulmonary and GI infections such as *Klebsiella pneumoniae*, *Streptococcus pneumoniae*, *Salmonella enterica* ser. Typhimurium and *Clostridium difficile* [18–21]. In animal infection models using these pathogens, IL-22 primarily plays a protective role by maintaining barrier integrity and thereby limiting bacterial dissemination. This is achieved via an IL-22-mediated increase in proliferation and inhibition of apoptosis of epithelial cells and stem cells [22–24]. IL-22 also increases production of protective mucins and antimicrobial peptides [25, 26]. Although the role of IL-22 in *B*. *anthracis* infection has not been reported, the cytokine could play a similar role in both pulmonary and GI anthrax infection.

Regulation of IL-22 production by ILC3s is controlled through several well-described signaling pathways. IL-23-mediated activation of the STAT3 signaling pathway is essential for IL-22 production [27]. The other major transcription factor involved in IL-22 production is the aryl hydrocarbon receptor (AHR), which is important not only for *Il22* expression in ILC3s [28], but also for the development of ILC3s from immature precursors [29]. STAT3 and AHR are important in the analogous adaptive immune system T helper 17 (Th17) subset [30]. Because of the relatedness of ILC3s and Th17 cells, there are likely unidentified signaling pathways that contribute to IL-22 regulation in ILC3s.

Lethal toxin is a well-characterized two-subunit bacterial toxin from *B*. *anthracis* comprised of protective antigen and lethal factor [31, 32]. Together the two proteins form a complex that binds to one of two known cell surface receptors, mediating entry into the host cell [31]. In the cytoplasm, lethal toxin is a zinc metalloprotease that cleaves a number of identified proteins, including members of mitogen-activated protein kinase (MAPK) signaling pathways [33] and the inflammasome-activating sensor NLRP1B [34]. Lethal toxin effects are best described for macrophages and DCs, where its interference in MAPK signaling leads to reduced antigen presenting cell function, resulting in reduced T cell responses [35]. Lethal toxin also directly affects the function of T cells, B cells and NK T cells [36–39]. Whether lethal toxin impacts the function of ILCs has not been investigated. Here we show that lethal toxin impairs IL-22 production by ILC3s using *in vitro* and *in vivo* studies. Mechanistically, we identified previously unknown molecular circuits involving MAPK signaling that regulate IL-22 production in ILC3s. These data have implications on strategies to modulate either the function of ILC3 or the biological activity of IL-22 during infectious disease or inflammation.

## Results

### Lethal toxin down-regulates human and mouse IL-23-mediated production of IL-22 in ILC3s

**Fig 1:**
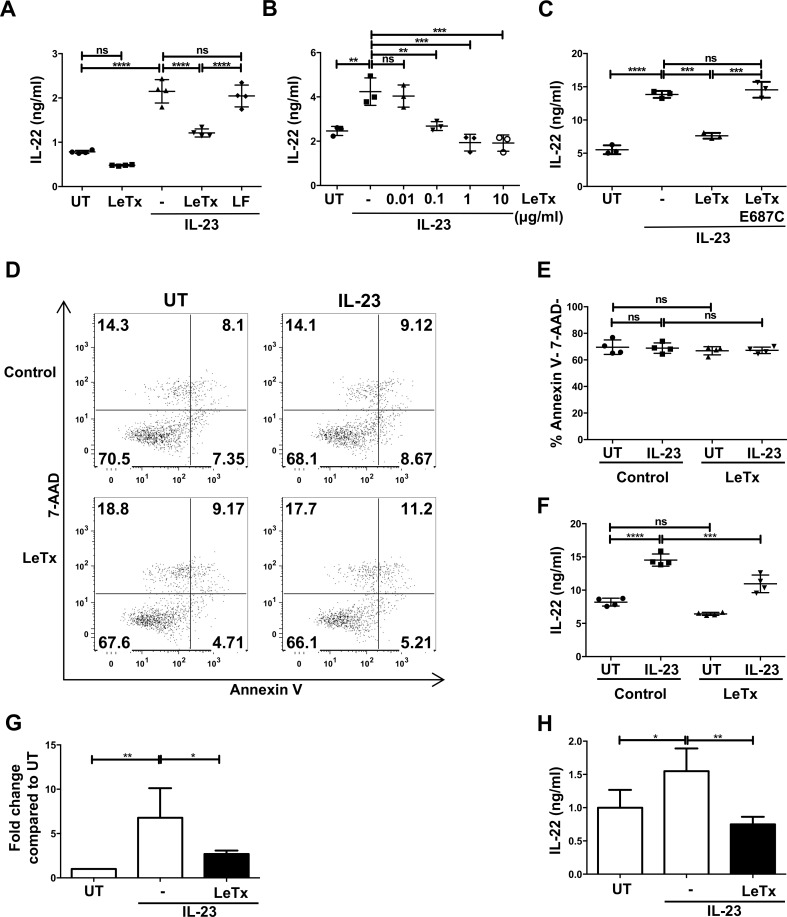
Lethal toxin down-regulates mouse IL-23-mediated production of IL-22 in ILC3s. (**A-C**) *Rag1*^*-/-*^ splenocytes were pretreated with (A) either lethal toxin (LeTx, lethal factor + protective antigen) or lethal factor (LF) (1 μg/ml) or (B) increasing doses of lethal toxin or (C) with enzymatic mutant toxin (E687C) for 3 hr followed by IL-23 (50 ng/ml) stimulation for 18 hr. Cell supernatants were analyzed for IL-22 secretion by ELISA. (**D-F**) *Rag1*^*-/-*^ splenocytes were treated with lethal toxin followed by IL-23 stimulation for 6 hr. Cell death was assessed by Annexin V and 7-AAD staining. (D) Shown are representative plots from one experiment of three. (E) Quantified apoptosis data and (F) IL-22 secretion from the same experiment are shown. (**G-H**) CD127^+^ ILCs were purified from spleens of *Rag1*^*-/-*^ mice and subjected to lethal toxin treatment as in A and measured for (G) *Il22* mRNA expression and (H) IL-22 secretion after 6 hr of IL-23 stimulation. Shown are mean±SD from one experiment of at least three experiments (A-F). Shown are mean±SD from five experiments (G-H). * p≤0.05, ** p≤0.01, *** p<0.001, **** p<0.0001 and non-significant (ns) p>0.05 by one-way ANOVA with Tukey’s post-hoc test.

We used *Rag1*^*-/-*^ mouse splenocytes, a source of ILC3s, because the absence of T cells in this model makes ILC3s the dominant IL-22-expressing cell type [40]. Cells were treated with or without lethal toxin (lethal factor and protective antigen) for 3 hrs and then stimulated with or without IL-23 to induce IL-22 expression. As expected, IL-23 stimulation resulted in 2-fold greater amounts of secreted IL-22 (Fig 1A). Lethal toxin treatment reduced IL-23-mediated IL-22 levels by approximately 40% compared to non-toxin treated controls (Fig 1A). This decrease occurred in a lethal toxin dose-dependent manner (Fig 1B) and was dependent on enzymatically active lethal factor (Fig 1C), which was capable of gaining cell entry via protective antigen-mediated translocation (Fig 1A).

The term lethal toxin is a misnomer and the toxin has limited ability to kill most cells [41]. Nevertheless, we examined whether the reduction of IL-22 production by ILC3s correlated with a decrease in cell viability. Using two different viability exclusion dyes via flow cytometry analysis, we observed no difference in the viability of ILCs treated with or without lethal toxin and/or IL-23 (Fig 1D, 1E and 1F and S1A and S1B Fig). Thus, lethal toxin-mediated down-regulation of IL-22 was not due to reduced cell viability and instead suggested interference with a signaling pathway that regulates IL-22 production.

Reduced levels of secreted IL-22 in heterogeneous lymphocyte mixtures of *Rag1*^*-/-*^ splenocytes could be due to another cell affecting ILC3 production of IL-22 and/or modulation in IL-22 from other innate immune cells that have been reported to produce low levels of IL-22, such as DCs or neutrophils [22, 42, 43]. The ILCs present in *Rag1*^*-/-*^ splenocytes bound protective antigen in a dose-dependent manner (S1C Fig), suggesting the cells were direct targets for lethal toxin. To examine IL-22 production specifically in ILCs, we isolated ILCs through cell sorting from *Rag1*^*-/-*^ mice. Purified mouse ILCs (Lin^-^ CD127^+^, which may include ILC1s, ILC2s and ILC3s) were treated with or without lethal toxin and/or IL-23 and then *Il22* expression and IL-22 production were examined 6 hr later. Lethal toxin down-regulated IL-23-mediated *Il22* expression at the transcriptional level (Fig 1G). As we found in the heterogeneous splenocyte cultures, lethal toxin down-regulated secreted IL-22 (Fig 1H). These data indicate that lethal toxin modulated *Il22* and IL-22 at both the mRNA and protein levels, respectively, in ILCs and this was caused by a direct intoxication of ILC3s.

**Fig 2:**
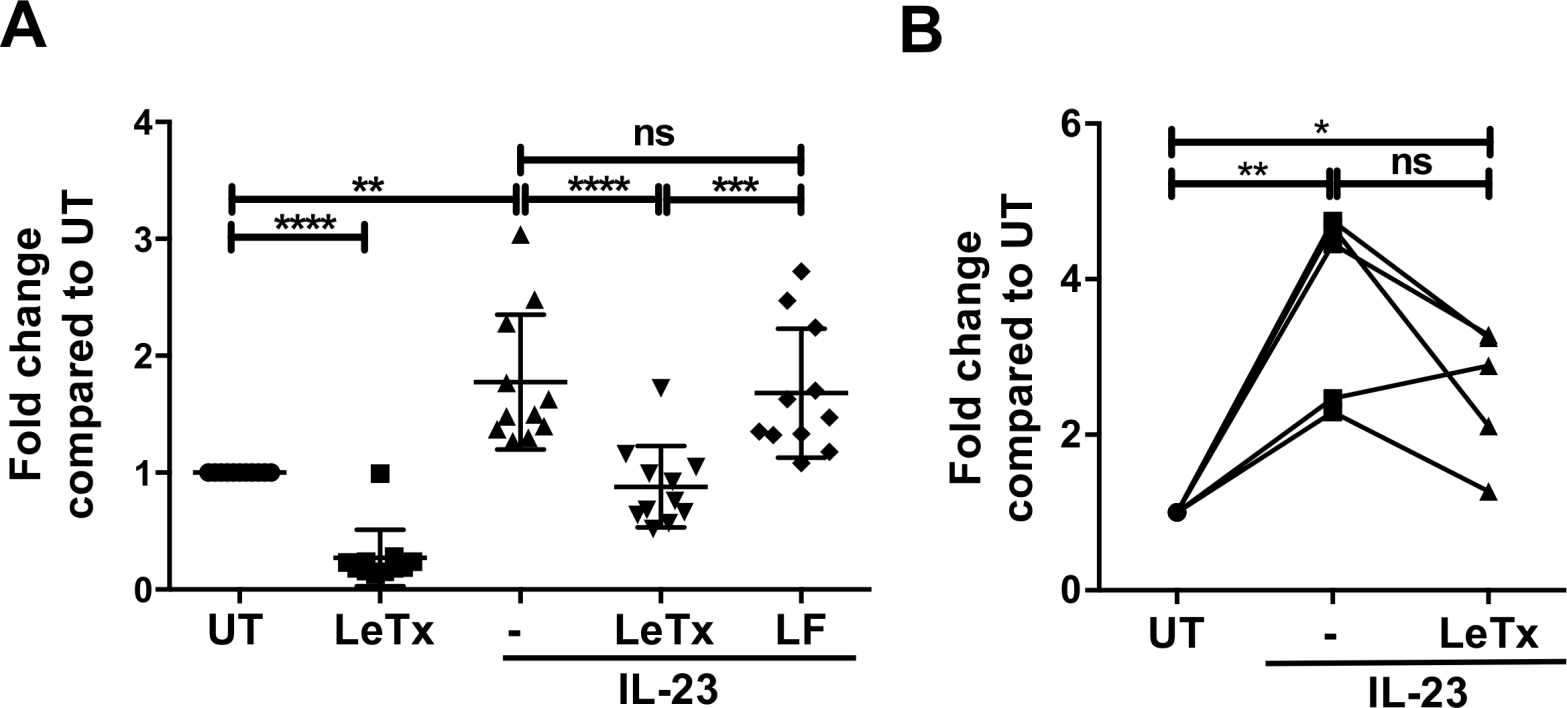
Lethal toxin decreases IL-22 production in human ILCs. (**A**) Human tonsillar lymphocytes or (**B**) sorted Lin^-^ CD127^+^ cells were pretreated with lethal toxin (1 μg/ml) for 2–3 hr followed by IL-23 (50 ng/ml) stimulation for 18 hrs. Cell supernatants were analyzed for IL-22 by ELISA. Shown is mean±SD of at least 10 donors (A) or 5 donors (B). * p≤0.05, ** p≤0.01, *** p<0.001, **** p<0.0001 and non-significant (ns) p>0.05 by one-way ANOVA with Tukey’s post-hoc test.

We next examined whether lethal toxin down-regulated IL-22 production in human ILCs. Using a mixed lymphocyte population obtained from a mucosal tissue (tonsils) we treated cells with or without lethal toxin for 3 hr and then stimulated the cells with or without IL-23. As for mouse ILC3s, low levels of secreted IL-22 were detected in human lymphocyte cultures, which significantly increased by approximately 2-fold after IL-23 stimulation (Fig 2A). Lethal toxin treatment prevented an induction of IL-22 levels by IL-23, which depended on toxin translocation since lethal factor (LF) alone failed to inhibit IL-22 production (Fig 2A). The inhibition by lethal toxin was dose-dependent and required enzymatically active toxin (S2A and S2B Fig). We also examined the effects of lethal toxin on purified human ILCs (Lin^-^ CD127^+^) and found that IL-23-mediated IL-22 secretion was generally decreased in the presence of the toxin (Fig 2B). In summary, lethal toxin down-modulated IL-23-mediated IL-22 production in both mouse and human ILC3s. These findings are consistent with the notion that lethal toxin inhibits IL-23 signal transduction leading to IL-22 production in ILC3s.

### Lethal toxin has no detectable effects on IL-23-mediated STAT3 activation in ILC3s

**Fig 3:**
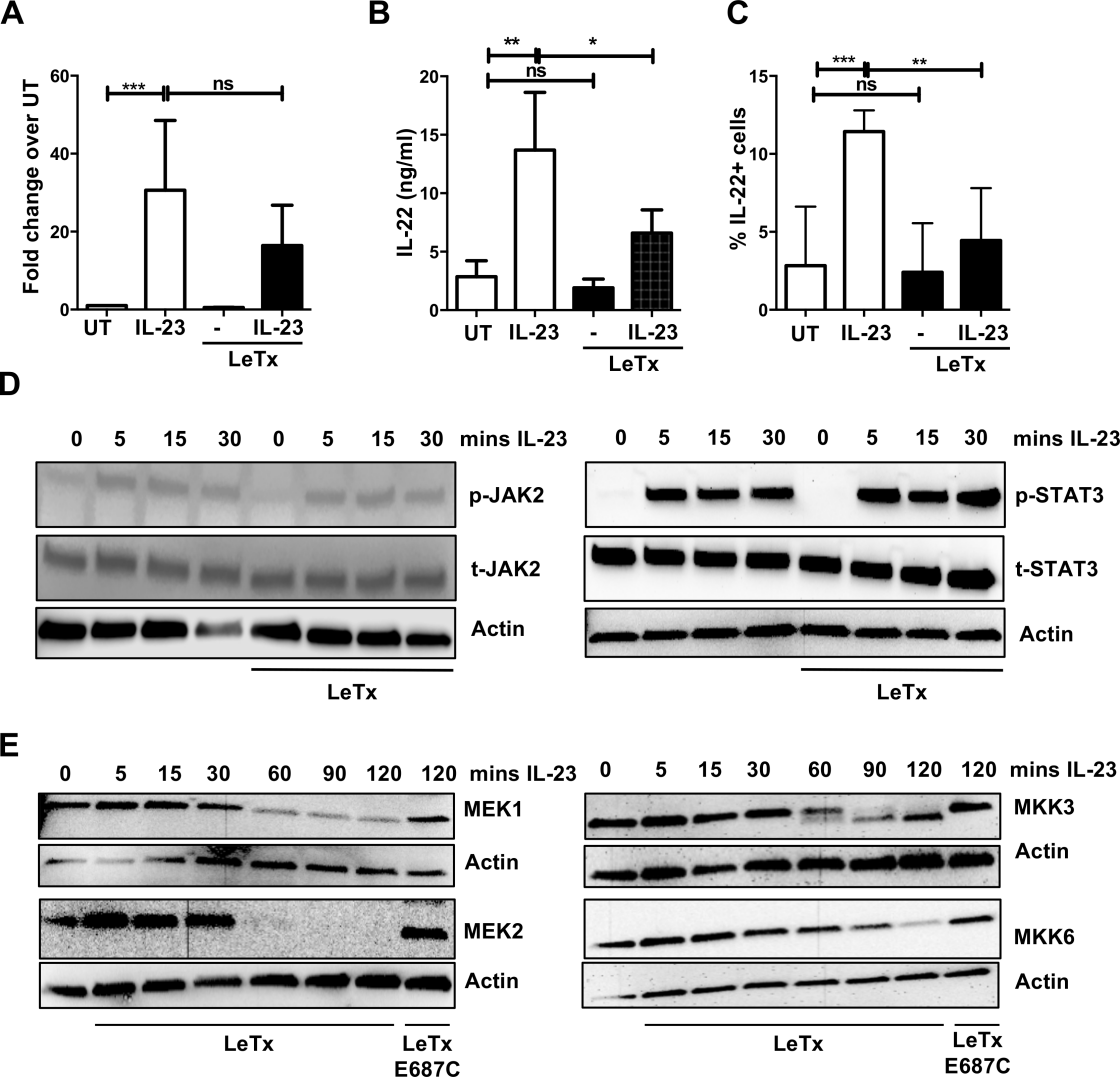
Destabilization of MEKs by lethal toxin in ILC3s. MNK-3 cells were pretreated with lethal toxin for 2 hr followed by IL-23 stimulation for 5–6 hr. (**A**) *Il22* expression was analyzed by real time RT-PCR (n = 5), (**B)** secreted IL-22 in supernatants was quantitated by ELISA (n = 4) and (**C**) the percent cells producing IL-22 (%IL-22^+^) was determined by intracellular cytokine staining (n = 3). * p≤0.05, ** p≤0.01, *** p<0.001, **** p<0.0001 and non-significant (ns) p>0.05 by one-way ANOVA with Tukey’s post-hoc test. (**D**) The JAK2-STAT3 pathway was not affected by lethal toxin in MNK3 cells. MNK3 clone B3 cells were treated with lethal toxin for 2 hr followed by IL-23 stimulation for the time points indicated. Levels of phosphorylated JAK2 (p-JAK2), total JAK2, phosphorylated STAT3 (p-STAT3), total STAT3 and actin were analyzed by immunoblot analysis. Shown are representative data from one experiment of 2–3 independent experiments. (**E**) MNK-3 cells were treated with lethal toxin or mutant lethal toxin (E687C) (1 μg/ml) for time points indicated. Levels of MEK1, MEK2, MKK3, MKK6 and actin were analyzed by immunoblot analysis. Shown are representative data from one experiment of 2–3 independent experiments.

Recognition of IL-23 by its cell surface receptor on cells primarily activates JAK2 and the downstream transcription factor STAT3 [44]. To determine if lethal toxin had any effect on STAT3 signaling, we tested the effect of the toxin on JAK2 and STAT3 phosphorylation. As primary ILC3s are rare and difficult to purify in sufficient quantities, we made use of a recently generated ILC3 cell line, MNK-3, that was isolated from mouse fetal thymic immune precursors [45]. These cells produce high levels of IL-22 upon stimulation with IL-23 and their use permitted both semi-quantification of transcript expression and visualization of cellular protein and phospho-protein levels by western blot. We found that lethal toxin inhibited both constitutive and IL-23-mediated *Il22* expression and IL-22 production in this cell line (Fig 3A, 3B and 3C) without affecting cell viability (S3A–S3C Fig), recapitulating our experiments with primary mouse and human cells. To examine whether lethal toxin modulated JAK2 and/or STAT3 signaling, we next treated MNK-3 cells with lethal toxin for 2 hr, stimulated with IL-23 for the indicated time and then examined JAK2 and STAT3 phosphorylation by western blot analyses. We found that lethal toxin treatment had no detectable effect on the levels of phosphorylated JAK2 or STAT3 after IL-23 treatment (Fig 3D). Thus, lethal toxin did not interfere with the JAK-STAT pathway, the canonical IL-23 signaling pathway in ILC3s.

### Destabilization of MEK kinases by lethal toxin in ILC3s

In other immune cells, particularly macrophages, lethal toxin is known to cleave MEK1 and MEK2 and lead to degradation of these proteins [33]. Thus we also examined MEK1 and MEK2 levels in MNK-3 cells after incubation with lethal toxin. Treatment of MNK3 cells with lethal toxin, but not toxin lacking enzymatic activity, led to a time-dependent reduction in MEK1 and MEK2 levels (Fig 3E). Lethal toxin has also been reported to affect other MEK kinases, including MKK3 and MKK6 [46, 47]. Therefore, we also examined MKK3 and MKK6 levels in MNK-3 cells after lethal toxin treatment. We found that lethal toxin led to reduced levels of MKK3 and MKK6, albeit with slower kinetics than that observed for MEK1 and MEK2 (Fig 3E). Together, these data show that lethal toxin treatment leads to loss of MEK1, MEK2, MKK3 and MKK6 in ILC3-like MNK-3 cells. These data are also consistent with lethal toxin mediating the degradation of these proteins.

### Down-regulation of downstream of MEK effector pathways in ILC3s by lethal toxin

**Fig 4:**
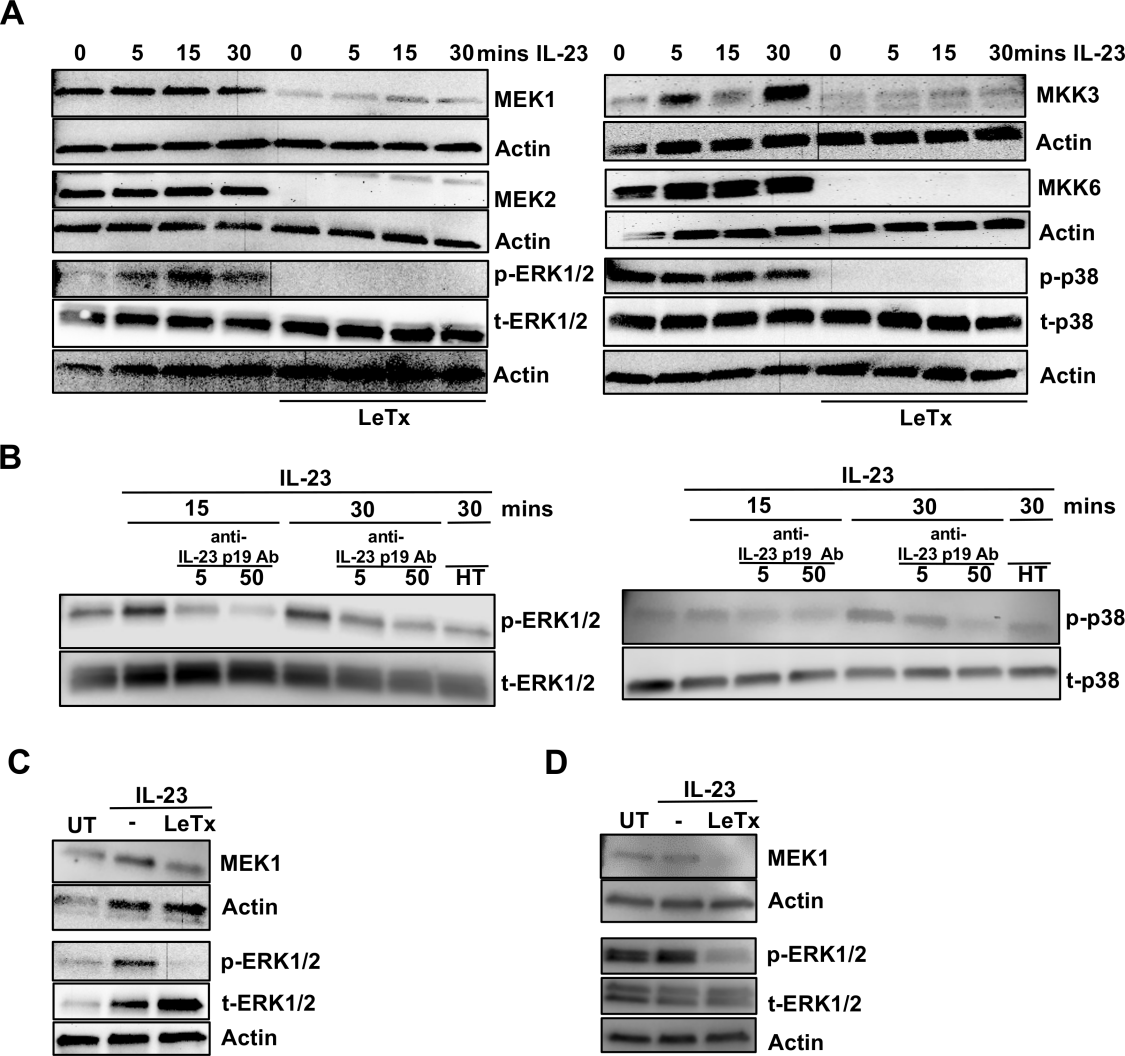
Down-regulation of ERK1/2 and p38 activation in ILC3s by lethal toxin. MNK-3 cells were pretreated with lethal toxin (1 μg/ml) for 2 hr followed by IL-23 stimulation as indicated. Cell lysates were harvested and subjected to immunoblot analysis for MEK1, MEK2, MKK3, MKK6, phosphorylated ERK1/2 (p-ERK1/2), total ERK1/2 (t-ERK1/2), phosphorylated p38 (p-p38), total p38 (t-p38) and actin. Shown is a representative blot for n = 2–4 experiments. **(B)** MNK-3 cells were pretreated with anti-IL-23 p19 antibody (5 or 50 μg/ml) for 1 hr followed by IL-23 stimulation for 15 or 30 min. Cell lysates were subjected to immunoblot analysis for phosphorylated ERK1/2 (p-ERK1/2), total ERK1/2, phosphorylated p38 (p-p38), total p38 and actin. Shown is a representative blot for 2–4 experiments. (**C**) *In vitro* expanded human Lin^-^ CD127^+^ cells or (**D**) mouse Lin^-^ CD127^+^ c-Kit^+^ Thy1.2^+^ cells expanded from splenocytes of *Rag1*^*-/-*^ mice were treated with lethal toxin for 2 hr followed by IL-23 stimulation for 30 mins. Cell lysates were analyzed for MEK1, phosphorylated ERK1/2 (p-ERK1/2), total ERK1/2 and actin by immunoblot analysis. Shown are representative blots from 2 independent experiments for both C and D.

MEK1/2 and MKK3/6 are MAPK kinases upstream of several MAPK pathways, including ERK1/2 and p38, respectively, and have well described roles in many immune cells [48], but this has not been studied in IL-23-stimulated ILC3s. IL-23 treatment of MNK-3 cells led to temporal phosphorylation of ERK1/2 (Fig 4A). ERK1/2 activation was rapid, and could be detected within 5 min of IL-23 addition, suggesting a direct activation of ILC3s by IL-23. This activation was dependent on intact IL-23, as heat-treated (HT) IL-23 was unable to phosphorylate ERK1/2 (Fig 4B). Furthermore, neutralization of IL-23 inhibited IL-23-mediated ERK1/2 phosphorylation (Fig 4B). Importantly, the phospho-ERK1/2 signal was completely abrogated upon lethal toxin treatment, which also correlated with loss of MEK1 and MEK2 (Fig 4A).

In addition to the mouse cell line, we also examined whether IL-23 treatment results in ERK1/2 phosphorylation in human ILC3s. As these are very rare cells, we first expanded primary sorted ILCs *in vitro* to obtain larger numbers of ILCs (S4 Fig). IL-23 stimulation of the expanded human ILCs led to increased phosphorylation of ERK1/2, which did not occur when the cells were treated with lethal toxin (Fig 4C). Similar results were obtained with expanded mouse ILCs (Fig 4D). Thus, IL-23 directly activates the ERK1/2 signaling pathway in ILC3s and this activation is inhibited by lethal toxin.

Unlike IL-23-mediated ERK1/2 activation, p38 activation was not modulated by IL-23 but it was modulated by lethal toxin in MNK-3 cells. Phosphorylated p38 was found in unstimulated MNK-3 cells, suggesting basal levels of p38 activation in cells that is not readily increased by IL-23 stimulation (Fig 4A). However, this phosphorylated p38 was undetectable in lethal toxin treated cells (Fig 4A), correlating with lack of MKK3 and MKK6, suggesting the toxin interfered with p38 activation through upstream interference of MKK levels. As p38 activation is often associated with IL-1β signaling, and IL-1β can also induce IL-22 in ILC3s, we also examined the effect of lethal toxin on IL-1β-mediated activation of ILC3s. We found that lethal toxin suppressed IL-22 production (S5A and S5B Fig) and also ablated IL-1β-mediated phoshphorylation of p38 through loss of total p38 (S5C Fig). Together, these data show that treatment of ILC3s with lethal toxin prevented activation of ERK1/2 and the maintenance of p38, which correlated with the loss of their upstream activators, MEK1/2 and MKK3/6.

**Fig 5:**
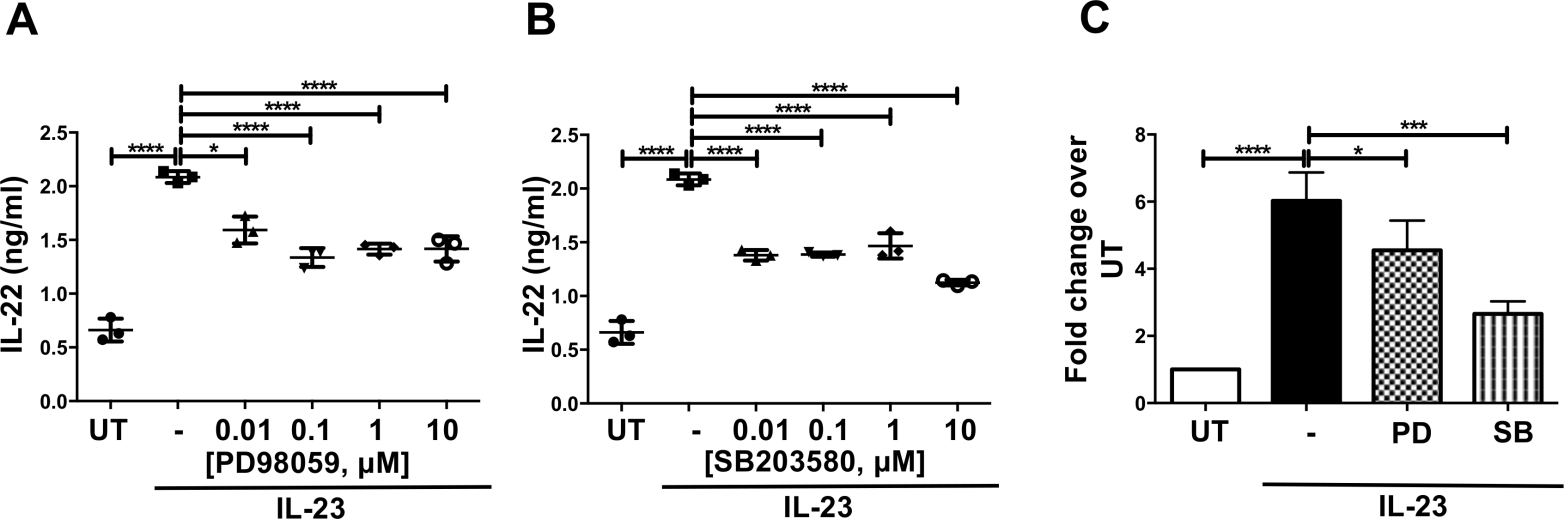
Inhibition of p38 signaling down-regulates IL-23-mediated IL-22 production in ILC3s. *Rag1*^*-/-*^ splenocytes were pretreated with DMSO or indicated concentration of (**A**) PD98059 (MEK1 inhibitor) or (**B**) SB203580 (p38 MAPK inhibitor) for 1 hr followed by IL-23 stimulation for 18 hr. IL-22 secretion was examined by ELISA. **(C)** Sorted Lin^-^ CD127^+^ cells from *Rag1*^*-/-*^ splenocytes were pretreated with DMSO or indicated concentrations of PD98059 or SB203580 as in A and analyzed for IL-22 secretion by ELISA. In panel C shown is mean±SD from 3 experiments. * p≤0.05, ** p≤0.01, *** p<0.001, **** p<0.0001 and non-significant (ns) p>0.05 by one-way ANOVA with Tukey’s post-hoc test.

### Inhibition of p38 signaling down-regulates IL-22 production in ILC3s

As lethal toxin treatment led to reduced levels of MEK1/2 and MKK3/6 and reduced phosphorylation of ERK1/2 and p38, we examined the role of these two MAPK kinases in IL-22 production in ILC3s. To this end, we made use of two widely used small molecule inhibitors, PD98059 and SB203580. PD98059 is a selective inhibitor of MEK1/2 and therefore has downstream indirect inhibition of ERK1/2 [49] and SB203580 selectively inhibits p38 activation [50]. Cells were treated with an inhibitor or vehicle only control and then were stimulated with IL-23. Both inhibitors significantly reduced IL-23-mediated IL-22 production from *Rag1*^*-/-*^ splenocytes (Fig 5A and 5B). When we examined IL-22 secretion from purified mouse ILCs, we found that SB203580 down-regulated IL-23-mediated IL-22 production by approximately 50% whereas the effect of PD98059 was a reduction of 25% (Fig 5C). This indicated that between the two MAPK pathways, p38 may be the more important for IL-22 production in ILC3s. Thus, the p38 pathway is a positive regulator of IL-23-mediated IL-22 production in ILC3s whereas the ERK1/2 pathway appears to play a minor role.

### *In vivo* administration of lethal toxin alters *ex vivo* IL-22 and GM-CSF production by ILC3s

Our results suggest that lethal toxin from *B*. *anthracis* subverts ILC3 function by modulating the p38 signaling pathway. This finding may have implications for the host immune response or barrier function during infection. To translate our *in vitro* studies in mouse and human ILC3s to an intact organism, we examined if lethal toxin modulated ILC3 function in mice. Mice were intravenously injected with 100 ug each of lethal factor and protective antigen and then 48 hr later we examined the numbers of ILC3s in different tissues as well as the capacity of these ILCs to produce IL-22 or GM-CSF *ex vivo*. *Rag1*^*-/-*^ mice were used in order to examine ILCs in the absence of IL-22-producing T cells.

**Fig 6:**
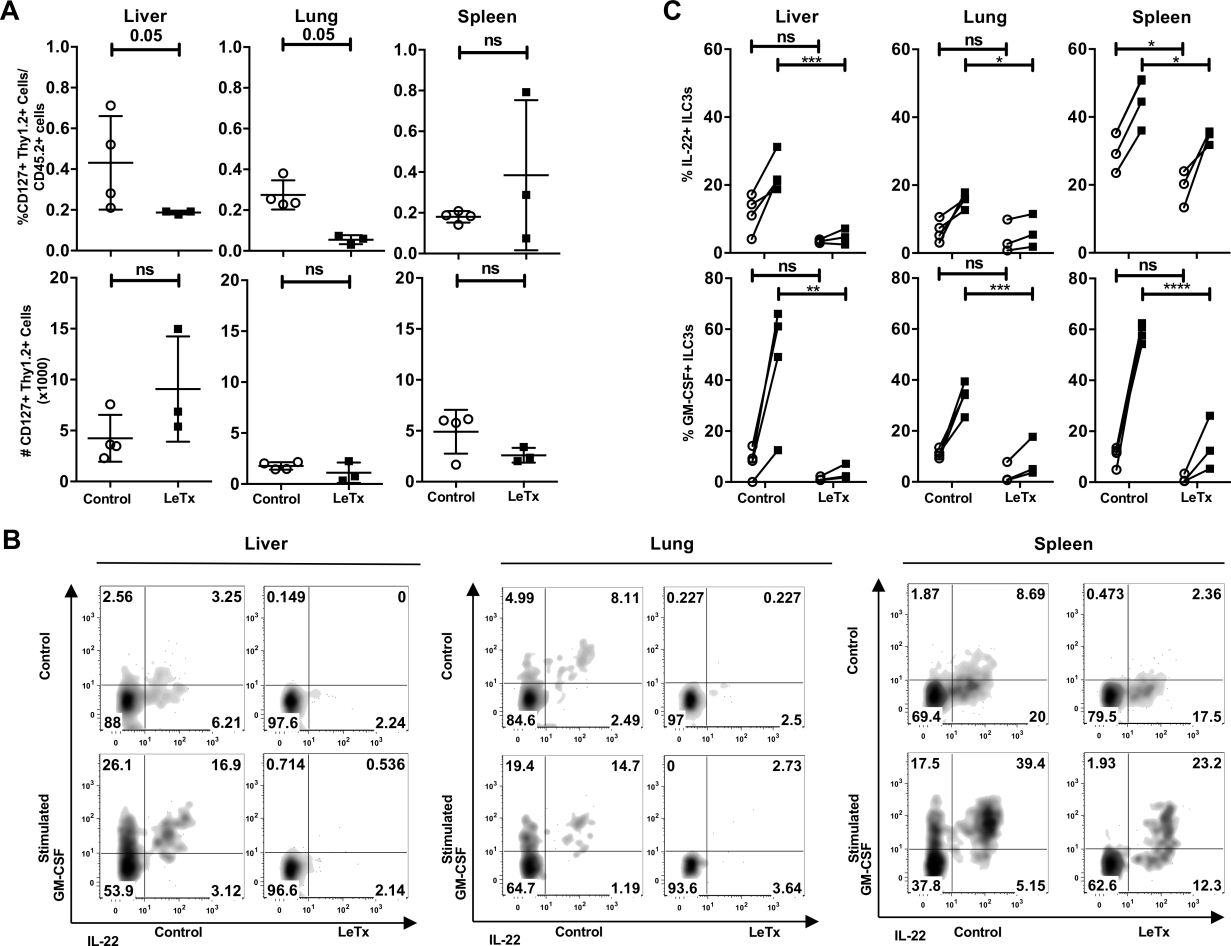
*In vivo* administration of lethal toxin decreased IL-22 and GM-CSF production in ILC3s. *Rag1*^*-/-*^ mice were administered control solution or 100 μg of lethal toxin by tail vein injection. Two days post-injection, mice were euthanized and (**A**) the percentage and total number of ILC3s in livers, lungs and spleens from control mice (open circles) or lethal toxin treated mice (closed squares) were determined by flow cytometry. (**B-C**) Isolated lymphocytes were unstimulated (open circles) or stimulated with IL-23, PMA and ionomycin (black squares) in the presence of brefeldin A for 5 hrs. IL-22 and GM-CSF production was determined by intracellular flow cytometry. Shown is a representative experiment of 2 experiments with 3 mice per group. * p≤0.05, ** p≤0.01, *** p<0.001, **** p<0.0001 and non-significant (ns) p>0.05 by Mann-Whitney test (A) and two-way ANOVA corrected for multiple comparison using Sidak’s multiple comparison test (C).

We found no change in the total number of ILCs (Lin^-^ CD45.2^+^ Thy1.2^+^ CD127^+^) in the spleens, lungs or livers of toxin treated mice compared to mice that were injected with vehicle control (Fig 6A and S6A Fig). To examine the capacity of these cells to produce cytokine upon *ex vivo* stimulation, we stimulated the cells with IL-23, PMA and ionomycin and examined IL-22 production by intracellular cytokine staining (Fig 6B and S6B Fig). In *ex vivo* culture, a low percentage of ILCs produced IL-22. Upon IL-23 stimulation, ILCs isolated from untreated mice had a greater percentage of cells expressing IL-22 than lethal toxin treated mice (Fig 6C). These data suggest that lethal toxin *in vivo* negatively modulates ILC3 function by decreasing the capacity of ILC3s to produce the key cytokine, IL-22.

In addition to IL-22, ILC3s also secrete other effector molecules, including the cytokine GM-CSF. In our *ex vivo* stimulated cells we also examined whether lethal toxin modulated GM-CSF production, which is induced in ILC3s by IL-1β [51]. Most ILCs that produced IL-22 also produced GM-CSF, but not all GM-CSF expressing cells produced IL-22 (Fig 6B). Significantly fewer ILCs from mice treated with lethal toxin produced GM-CSF compared to ILCs from control mice (Fig 6B and 6C). Therefore, *in vivo* lethal toxin administration can reduce the ability of ILC3s in several different tissues to produce IL-22 and GM-CSF.

## Discussion

We have found that *B*. *anthracis* lethal toxin down-regulated IL-23-mediated *Il22* expression and IL-22 secretion *in vitro* and IL-22 production in ILC3s *ex vivo*. Lethal toxin had no effects on the canonical IL-23 signaling pathway, via JAK2/STAT3, but did mediate loss of MEK1/2 and MKK3/6 from ILC3s. Reduced MEK1/2 and MKK3/6 levels correlated with reduced activation of the downstream MAPK signaling molecules ERK1/2 and p38, respectively. Experimental inhibition of MEK signaling pathways in ILC3s likewise resulted in reduced IL-22 production. Thus, MAPK signaling pathways, particularly the MKK3/6-p38 axis, are important in IL-23-mediated IL-22 production in ILC3s. Our studies were performed using mouse and human immune cells and included cell lines, primary cells and expanded primary cells providing robust results. Thus, our data are complementary and show that MAPK is a central signaling pathway in IL-23-mediated IL-22 production in ILC3s, suggesting that this is an evolutionarily conserved and important pathway.

MAPK are important for mediating signaling of IL-1β- or RET receptor-mediated IL-22 production, but have not yet been identified in IL-23-mediated signaling in ILC3s [15, 52]. Activation of multiple signaling pathways may allow cells to finely tune expression of effector molecules as well as provide redundancy and resiliency. During infection this allows immune responses to better counteract the virulence factors produced by pathogens.

Bacterial toxins are valuable molecular tools for elucidating signaling pathways in cells [53]. By probing the biology of *B*. *anthracis* lethal toxin we have identified MEK1/2-ERK1/2 and MKK3/6-p38 as important signaling pathways in the production of IL-22 in ILC3s. We found that IL-23 stimulation of ILC3s led to rapid phosphorylation of ERK1/2, suggesting a direct effect of IL-23 on ERK1/2 activation. ERK1/2 and p38 phosphorylation was inhibited in the presence of lethal toxin, which we showed caused loss of the upstream MAPK kinases, MEK1/2 and MKK3/6 [33, 46, 47]. IL-23 signaling strongly activates the STAT3 signaling pathway, which is critical in ILC3s for IL-22 production [27]. However, other signaling pathways, including MAPK, are less well understood. There is one report of MEK1/2 inhibitors modulating IL-23 signaling in DCs [54]; however, MAPK signaling is not a well-appreciated IL-23-mediated pathway in ILC3s.

ILC3s are rare, but can nevertheless have critical roles in the early immune response to pathogenic bacteria at mucosal barriers. Experimental elimination of these cells in a variety of bacterial infections worsens infection, especially in the absence of adaptive immunity. Mice lacking ILCs are more susceptible than control mice to such pathogenic bacteria as *Citrobacter rodentium* or *K*. *pneumoniae* [40, 55]. Less is known regarding the role of innate lymphocytes in *B*. *anthracis* infection. One study found that in a model of *B*. *anthracis* gastrointestinal infection that colonic ILC2s, a Th2-like innate lymphocyte subset with some similarities to ILC3s, were reduced in number [56]. For the ILC2s that did persist, a smaller percent produced the key ILC2 cytokines IL-5 and IL-13. Lethal toxin was shown to decrease the cytotoxicity of natural killer (NK) cells [57]. Combined with our data on ILC3s, it appears that *B*. *anthracis* modulates many subsets of innate lymphocytes, potentially through conserved mechanisms.

Lethal factor directly effects endothelial and epithelial cells to reduce barrier integrity during infection [5]. We have shown here another potential mechanism for how lethal toxin perturbs barrier function through the down-regulation of a key effector molecule, IL-22, in mucosal barrier maintenance and wound repair. Bacterial toxins are a key virulence factors in that modulate host signaling pathways [41]. Pathogenic bacteria and viruses have evolved strategies to disarm the innate immune system during infection [3]. As secreted proteins, bacterial toxins can have far-reaching effects within the host. In our *in vivo* toxin model we examined the systemic effects of lethal toxin on ILC3s. ILC3s isolated from the lungs, livers and spleens of lethal toxin treated mice were found to have reduced capacity to produce IL-22 and GM-CSF. This finding has important implications on the ability of the host to prevent bacterial dissemination and reduce and/or eliminate pathogen burden.

IL-22 is one of the most important effector molecules produced by ILC3s. IL-22 signaling in non-hematopoietic cells up-regulates expression of antimicrobial peptides, such as β-defensin, lipocalin-2, RegIIIβ, and RegIIIγ [20, 25, 58]. These antimicrobial peptides help regulate the commensal flora that helps prevent pathogen colonization, as well as directly counteract pathogens [59]. IL-22 is a potent inducer of mucins, the large glycoproteins that create a thick, often impenetrable barrier, on mucosal surfaces [60, 61]. IL-22 also plays a role in epithelial cell repair and has direct effects on the intestinal stem cells [22–24]. Mice deficient in IL-22 or ILC3s are more susceptible to many different bacterial infections, especially mucosal-associated pathogens. The role of IL-22 has been studied in a similar spore-forming mucosal Gram-positive bacterium, *C*. *difficile*. IL-22 deficient mice are more susceptible to *C*. *difficile* infection [21] and both ILC1s and ILC3s are important in controlling infection [62]. Our results show that reduction in IL-22 production by a bacterial toxin is an excellent strategy on the part of the pathogen to establish infection in the host. Reduced IL-22 in both the inflammatory milieu and systemically would have far reaching effects on the effectiveness of the immune response to combat infection.

In addition to IL-22, ILC3s produce other effector molecules such as IL-17 and GM-CSF. IL-17 is produced mainly by pathogenic ILC3s associated with chronic inflammation and cytokine dysregulation [11] and we did not readily detect IL-17 in our studies. GM-CSF is produced by ILC3s after stimulation [51, 63]. It is a potent cytokine in the development, further activation and trafficking of macrophages, DCs and other innate cells. GM-CSF also promotes an inflammatory environment for the adaptive arm of the immune system, especially in Th17 cell biology [64, 65]. In bacterial infections, GM-CSF is important for neutrophil and macrophage influx to sites of infection [63, 66].

In this study we have shown that a bacterial toxin perturbs signaling pathways in ILC3s, down-modulating the innate immune response. Our data implicate p38 and MAPK in IL-23-mediated signaling in ILC3s, identifying a new pathway to target in the regulation of ILC3 function. Identification of IL-23-mediated MAPK signaling in IL-22 production may lead to new or co-opted therapeutics. IL-22 is likely a beneficial cytokine in *B*. *anthracis* infection, but in chronic inflammatory conditions such as inflammatory bowl disease or psoriasis, IL-22 can have pathogenic consequences [67, 68]. Understanding the environmental and molecular factors that regulate IL-22 will be essential for developing more focused approaches to target the biology of IL-22.

## Materials and methods

### Lethal toxin and signaling inhibitors

Recombinant protective antigen and wild-type lethal factor or lethal factor mutant (E687C) were obtained from List Biologicals (Campbell, CA) or BEI Resources (Manassas, VA). PD98059 and SB203580 were both from Cell Signaling Technology (Danvers, MA).

### Mice

*Rag1*^*-/-*^ mice on a C57BL/6 background were obtained from The Jackson Laboratory (Bar Harbor, ME) [69]. Mice were housed in an AAALAC-accredited *Helicobacter*-free rodent barrier facility. All studies were approved by the OUHSC Institutional Animal Care and Use Committee.

### Human ILC3s

Tonsils were received after being discarded from pediatric surgery at OU Children’s Hospital (Oklahoma City, OK). Institutional Review Board approval was obtained prior to the initiation of our studies.

### Cell lines

MNK-3 cell line [45] and clone B3 were maintained in DMEM (Corning; Tewksbury, MA) with 10% heat-inactivated FBS (Gemini Bio-Products; West Sacramento, CA), 2 mM GlutaGro (Corning), 1 mM sodium pyruvate (GE Healthcare HyClone; Logan, UT), 55 μM β-mercaptoethanol (Sigma), 10 mM HEPES (Corning), 50 μg/ml gentamycin (Amresco; Solon, OH), 100 U/ml penicillin (Gemini Bio-Products), 100 U/ml streptomycin (Gemini Bio-Products) and 10 ng/ml recombinant mouse IL-7 and IL-15 (eBioscience; San Diego, CA or Peprotech; Rocky Hill, NJ).

### Splenocyte preparation

Spleens were excised and single cell suspensions were made by disruption of the spleen on wire mesh using the plunge of a 3 ml syringe. Cells were centrifuged and the cell pellet was resuspended. Red blood cells were lysed in ACK lysis buffer (0.83% NH_4_Cl, 0.5% KHCO_3_, 0.5 μM EDTA) for 2 min and then neutralized with PBS or media. Cells were counted using trypan blue exclusion staining and a hemocytomer.

### Human lymphocyte preparation

Tonsillar lymphocytes were isolated by passing minced tonsil pieces (approximately 1 mm) through a 70 μM cell strainer and washed with HBSS supplemented with 100 U/ml penicillin, 100 U/ml streptomycin, 5 μg/ml gentamicin, and 0.5 μg/ml amphotericin B (Corning). Tonsillar lymphocytes were isolated by layering single cell suspension over a Ficoll gradient (GE Healthcare Life Sciences; Pittsburgh, PA). After isolating cells from the interface, which are mostly leukocytes, RBCs were lysed. Cells were then washed in HBSS for three times to remove contaminating Ficoll. Tonsillar lymphocytes were aliquoted and stored in liquid nitrogen until future use.

### *In vitro* lethal toxin assay with *Rag1*^*-/-*^ splenocytes or human tonsillar lymphocytes

100,000–500,000 splenocytes per well were incubated in a round bottom 96 well plate in IMDM media supplemented with 10% FBS, 100 U/ml pencillin, 100 U/ml streptomycin, IL-2 (20 ng/ml) and IL-7 (10 ng/ml). Human tonsillar lymphocytes (2x10^6^) were plated in 200 μl RPMI media supplemented with 10% FBS, 100 U/ml penicillin, 100 U/ml streptomycin, non-essential amino acids, sodium pyruvate, IL-2 (20 ng/ml), IL-7 (10 ng/ml) and IL-1β (20 ng/ml). For initial lethal toxin experiments splenocytes/tonsillar lymphocytes were cultured in media containing in 0.1% serum to minimize the effect of serum on lethal factor enzyme activity. After the initial period of lethal toxin treatment (3 hrs), serum was replenished to a full concentration of 10%. Cells after toxin treatment were stimulated with recombinant mouse or human IL-23 (50 ng/ml) (eBioscience) for 18 hrs. Cell supernatants were harvested by centrifugation at 1,500 rpm for 5 minutes and then used for the measurement of IL-22.

### *In vitro* lethal toxin assay with MNK-3 cells

MNK-3 cells (an ILC3 cell line) were cultured in MNK-3 media (DMEM (High glucose), 10% FBS, 100 U/ml penicillin, 100 U/ml streptomycin, 2 mM Glutagro, 1 mM sodium pyruvate, 10 mM HEPES, 55 μM β-mercaptoethanol, 5 μg/ml gentamicin, 10 ng/ml IL-7 and 10 ng/ml IL-15). For lethal toxin experiments MNK-3 cells were cultured in media containing 10 ng/ml IL-7. Cells were treated with lethal toxin for 2 hrs followed by IL-23 stimulation for 6 hrs. Cell lysates were analyzed for RNA and supernatants were analyzed for secreted IL-22 by ELISA.

### Protective antigen (PA) binding assay

Splenocytes were isolated from *Rag1*^*-/-*^ mice and cultured in IMDM (Corning) containing 0.1% FBS, 100 U/ml penicillin, 100 U/ml streptomycin and 2 mM glutamine supplemented with IL-2 (20 ng/ml) and IL-7 (10 ng/ml). Cells were incubated with control (no PA) or PA-AlexaFluor647 at increasing concentrations (0–10 μg/ml). Cells were incubated at 37°C for 2 hrs. Cells were washed with 1% BSA and stained with surface markers for ILC3. Cells were analyzed by flow cytometry.

### ELISA

Mouse or human IL-22 ELISAs (Antigenix America; Huntington Station, NY) were performed according to the manufacturer’s protocols.

### Intracellular cytokine staining and FACS analysis

Cell were stimulated as described for 5 hr in the presence of brefeldin A (eBioscience) for the last 4 hr. Intracellular cytokine staining was performed according to the manufacturer’s protocol with fluorophore conjugated mAbs to IL-22 (clone Il22JOP) or GM-CSF (clone MP1-22E9). Cells were analyzed on a Stratedigm S1200Ex flow cytometer (Stratedigm, San Jose, CA) and data were analyzed using FlowJo v.9.6 (Tree Star; Ashland, OR).

### Surface staining

Single cell suspensions were washed in PBS and then stained for 20–30 min on ice in the dark with fluorophore-labeled Abs (for a complete list see Tables 1 and 2) and fixable viability dye (eBioscience). Samples were washed with PBS with 1% BSA. Samples were either analyzed immediately or fixed with 2% paraformaldehyde (PFA) and stored at 4°C until analysis by flow cytometry.

**Table 1:** FACS- mouse antibodies.

Antigen	Clone	Fluorophore	Company	Catalog number
CD3e	145-2C11	FITC	eBioscience	11–0031
CD11c	N418	FITC	eBioscience	11–0114
B220	RA3-6B2	FITC	eBioscience	11–0452
NK1.1	PK136	FITC	eBioscience	11–5941
NK1.1	PK136	PE-Cyanine7	eBioscience	25–5941
CD45.2	104	PE-Cyanine7	eBioscience	25–0454
CD117	2B8	PE	eBioscience	12–1171
CD127	A7R34	APC	eBioscience	17–1271
CD127	A7R34	PE	eBioscience	12–1271
CD127	SB/199	PE	eBioscience	12–1273
CD127	eBioSB/199	PerCP-eFluor710	eBioscience	46–1273
GR-1	RB6-8C5	FITC	eBioscience	11–5931
CD90.2	53–2.1	eFluor450	eBioscience	48–0902
CD90.2	53–2.1	APC-eFluor780	eBioscience	47–0902
F4/80	BM8	APC	BioLegend	123115
IL-22	IL22JOP	APC	eBioscience	17–7222
GM-CSF	MP1-22E9	PE	eBioscience	12–7331

**Table 2:** FACS- human antibodies.

Antigen	Clone	Fluorophore	Company	Catalog number
CD3	SK7	PE	eBioscience	12–0036
CD11c	3.9	PE	eBioscience	12–0116
CD14	61D3	PE	eBioscience	12–0149
CD16	eBioCB16	PE	eBioscience	12–0168
CD19	SJ25C1	PE	eBioscience	12–0198
CD34	4H11	APC	eBioscience	17–0349
CD45	H130	PE-Cyanine7	eBioscience	25–0459
CD56	CMSSB	PE	eBioscience	12–0567
CD117	104D2	PerCP-eFluor710	eBioscience	46–1178
CD117	104D2	FITC	eBioscience	11–1178
CD127	eBioRDR5	PE-Cyanine7	eBioscience	25–1278
CD161	DX12	BV421	BD Biosciences	562615
CD161	HP-3G10	PerCP—Cyanine 5.5	Tonbo Biosciences	65–1619
CD161	HP-3G10	PerCP—Cyanine 5.5	eBioscience	45–1619
IL-22	IL22JOP	APC	eBioscience	17–7222
GM-CSF	BVD2-21C11	BV421	BD Biosciences	562930

### Viability staining

Apoptosis was monitored by Annexin V and 7-AAD staining following manufacturers’ protocols (BD Biosciences or BioLegend). Briefly, cells were treated or not with lethal toxin for time indicated. After 6 hr, cells were centrifuged and the supernatant was removed and stored. Cell pellets were washed with PBS. Cells were surface stained to identify ILCs along with a fixable viability dye eFluor780 (eBioscience). Cells were then stained with Annexin V and 7-AAD. Stained cells were immediately analyzed by flow cytometry.

### Cell sorting

For isolating mouse primary ILCs, single cell preparations of *Rag1*^*-/-*^ splenocytes were surface stained for NK1.1 and CD127. Cells were sorted as (CD127^+^ NK1.1^-^) and (CD127^-^ NK1.1^+^) using a FACSAria. For sorting human ILCs, tonsillar lymphocytes were depleted of B cells using CD19 magnetic beads (eBioscience). The B cell depleted fraction was then surface stained for lineage markers (CD3, CD19, CD14), CD127 and cells were sorted (Lin^-^ CD127^+^).

### *In vitro* expansion of human and mouse ILCs

Sorted human ILC3s (Lin^-^ CD127^+^) were cultured at 2,000–5,000 cells per well of a 96 well plate with irradiated (30 Gy) OP9-DL1 feeders [70]. Cells were cultured in RPMI with 10% FBS, 100 U/ml penicillin, 100 U/ml streptomycin, 1 mM sodium pyruvate, non-essential amino acids, 50 μM β-mercaptoethanol, 2mM glutamine and recombinant human IL-2 (20 ng/ml), IL-7 (20 ng/ml), SCF (20 ng/ml), FLT3L (10 ng/ml), IL-15 (10 ng/ml) for up to three weeks. For mouse ILC3s, splenocytes from *Rag1*^*-/-*^ mice were sorted (Lin^-^ (CD3, CD45R, CD11c, GR-1, NK1.1) c-kit^+^ Thy1.2^+^) and cultured as described for human cells using recombinant mouse cytokines.

### Real time RT-PCR

Cells were harvested in Trizol (Life Technologies; Carlsbad, CA) or TriPure (Roche; Nutley, NJ) and RNA was prepared according to the manufacturers’ protocols. RNA was DNase treated (Roche) and cDNA was reverse transcribed using Transcriptor (Roche) with oligo dT as the primer. cDNA was used as template in a real time PCR reaction using ABI Taqman primer-probes sets (Table 3) on a ABI 7500 Fast real time PCR machine (Life Technologies). cDNA was semi-quantitated using the ΔΔC_T_ method with *Hprt* (mouse) or *HPRT* (human) as an internal control for all samples.

**Table 3:** Applied biosystems primer probe sets (ThermoFisher).

Gene	Species	Catalog #
*Il22*	*Mus musculus*	Mm00444241_m1
*Hprt*	*Mus musculus*	Mm01545399_m1
*IL22*	*Homo sapiens*	Hs01574154_m1
*HPRT*	*Homo sapiens*	Hs02800695_m1

### Western blotting

After stimulation, cells were washed with PBS. Cells were lysed with TN1 lysis buffer (50 mM Tris, 125 mM NaCl, 1% Triton X-100, 10 mM EDTA, 10 mM sodium fluoride and 10 mM sodium pyrophosphate) supplemented with protease inhibitor cocktail (1.2 mM AEBSF, 0.46 μM aprotinin, 14 μM bestatin, 12.3 μM E-64, 112 μM leupeptin, 1.16 μM pepstatin) (Amresco; Solon, OH)) and 1 mM sodium orthovanadate (Enzo Life Sciences; Farmingdale, NY). Cell lysates were centrifuged at 15,000 rpm for 5 min. Cell supernatants were separated by SDS-PAGE on a 4–15% gradient gel (Bio-Rad; Hercules, CA). Proteins were transferred to an Immobilion-P PVDF membrane (EMD Millipore; Billerica, MA) using a wet transfer method. The protein-transferred membrane was blocked with 5% milk and then incubated with the manufacturers’ recommended concentration of primary antibody overnight at 4°C (Table 4). Blots were then washed and incubated with the appropriate species-specific-HRP secondary antibody (1:1000) for 1 hr. Blots were developed using Pierce ECL2 Western Blotting Substrate (Thermo Scientific; Waltham, MA) and imaged using a FluorChemQ (Alpha Innotech; San Leandro, CA). Images were semi-quantitated using open source Image J software. For reblotting with another antibody, blots were stripped using a Restore Western Blot Stripping Buffer (Thermo Scientific) and then washed and re-blocked and used as indicated above.

**Table 4:** Western blotting antibodies.

Antigen	Ab species	Company	Catalog number
JAK2	Rabbit	Cell Signaling Technology (Danvers, MA)	3230
Phosphorylated JAK2 (Tyr1007)	Rabbit	Cell Signaling Technology	4406
STAT3	Rabbit	Cell Signaling Technology	9139
Phosphorylated STAT3 (Tyr705)	Mouse	Cell Signaling Technology	9145
MEK1	Rabbit	Cell Signaling Technology	9146
MEK2	Rabbit	Cell Signaling Technology	9125
MKK3	Rabbit	Cell Signaling Technology	5674
MKK6	Rabbit	Cell Signaling Technology	8550
ERK1/2	Rabbit	Cell Signaling Technology	4695
Phosphorylated ERK1/2 (Thr202/Tyr204)	Rabbit	Cell Signaling Technology	4370
p38	Rabbit	Cell Signaling Technology	8690
Phosphorylated p38 (Thr180/Tyr182)	Rabbit	Cell Signaling Technology	4511
actin	Goat	Santa Cruz Biotechnology, Inc (Dallas, TX)	sc-1616
Rabbit IgG-HRP		Cell Signaling Technology	7074
Mouse IgG-HRP		Cell Signaling Technology	7076
Goat IgG-HRP		KPL	14-13-06

### *In vivo* toxin administration

Age- and sex-matched *Rag1*^*-/-*^ mice were injected intravenously with 100 μg lethal factor and 100 μg protective antigen in vehicle or as a control vehicle only via the lateral tail vein. Forty-eight hr later, mice were euthanized and lymphocytes were isolated from the spleens, livers and lungs. Cells were restimulated for 5 hr in the presence of BFA with or without 5 μg/ml phorbol 12-myristate 13-acetate (PMA) (Sigma), 0.5 μg/ml ionomycin (Sigma) and 50 ng/ml IL-23. Cells were then surface stained, subjected to intracellular cytokine staining and analyzed by flow cytometry as described above.

### Preparation of liver lymphocytes

Livers were excised and homogenized into a single cell suspension. Lymphocytes were isolated from the liver as previously described [71]. Liver homogenate was incubated with 100 U/ml collagenase (Sigma; St. Louis, MO) and 20 μg/ml DNase I (Sigma) for 40 min at 37°C. To remove hepatocytes, homogenates were centrifuged at 300 rpm for 3 min, and then supernatants were centrifuged at 1500 rpm for 10 min. The cells were resuspended in 1 ml complete media and 4 ml of 30% OptiPrep (Sigma) in a sodium phosphate buffer and 1 ml of media was carefully layered on top. Cells were centrifuged at 2,700 rpm for 20 min. The top layer and interface were harvested as the liver lymphocyte population.

### Preparation of lung lymphocytes

Lung lymphocytes were isolated as previously described [72]. Briefly, lungs were excised and mechanically homogenized into fragments of 1 mm. Homogenized tissue was transferred into 15 ml conical tube containing 5 ml of lung digestion medium (DMEM supplemented with 10% FBS, 100 U/ml penicillin, 100 U/ml streptomycin, 50 μM β-mercaptoethanol, 250 U/ml collagenase IV, 25 U/ml DNase I). Tissue was digested at 37°C for 20 mins. The digested tissues were pushed through a 70 μM cell strainer to generate a single cell suspension. The single cell suspensions were washed with serum free RPMI medium and centrifuged at 400Xg for 5 mins at 4°C. Cell pellets were then resuspended in 40% Percoll. Cells were then differentially centrifuged at 400Xg for 10 mins at 4°C without braking. Cell supernatants were discarded and cell pellets were lysed of RBCs and then used for staining with antibodies for flow cytometry analysis.

### Ethics statement

All animal experiments were conducted in accordance with the Animal Welfare Act and the recommendations in the Guide for the Care and Use of Laboratory Animals of the National Institutes of Health. The University of Oklahoma Health Sciences Center (OUHSC) animal facilities have full accreditation from the Association for Assessment and Accreditation of Laboratory Animal Care and are PHS-assured (Assurance Number: # A3165-01). All animal procedures were approved by the OUHSC Animal Care and Use Committee (IACUC) under protocol 14–094 and the Office of Animal Welfare Assurance (OAWA), which oversees the administration of the IACUC at OUHSC.

### Statistical analysis

Values are expressed as mean±SD. For two-way comparisons, a standard paired *t* test was used. For multiple comparisons, one-way ANOVA with Tukey’s post hoc analysis was used. For the *in vivo* experiment, two-way ANOVA corrected for multiple comparisons using Sidek’s multiple comparison test was used. Significance was defined as a value of *p*<0.05.

## Supporting information

S1 Fig Related to Fig 1 identification of ILC3s from mouse splenocytes, alternate viability assay and binding of protective antigen (PA) to ILC3s. **(A)** Gating strategy for the apoptosis assay is shown. Cells were gated on forward and side scatter followed by Lin (CD3, CD11c, B220, GR-1 and NK1.1)^-^ and Thy1.2^+^ CD127^+^ cells were analyzed for apoptosis by Annexin V and 7-AAD staining or for necrosis using viability eFluor780 dye. **(B)** Shown is a representative plot of viability eFluor780 stained samples (left) and the percentage of live cells (mean±SD, right) of a single representative experiment, shown in Fig 1D of 3 independent experiments. **(C)** Dose-dependent binding of mouse ILC3s (top) and RAW264.7, a macrophage cell line (bottom), and gating strategy for ILC3 of a representative experiment of 3 experiments is shown. Protective antigen (PA)-Alexa647 at indicated concentrations: 0 ug/ml (red), 0.01 μg/ml (blue), 0.1 μg/ml (green), 1 μg/ml (orange) and 10 μg/ml (cyan) were used to determine the binding to ILC3 or RAW264.7 mouse macrophages.
(PDF)

S2 Fig Related to Fig 2 lethal toxin decreases IL-22 production in human ILC3s in a dose- and enzymatic-activity dependent manner. **(A)** Lethal toxin decreased IL-22 production in a dose-dependent manner in human tonsillar lymphocytes. Human tonsillar lymphocytes were treated with increasing concentrations (0.01–10 μg/ml) lethal toxin for 3 hrs followed by IL-23 (50 ng/ml) stimulation for 18 hr. Cell supernatants were analyzed for IL-22 secretion by ELISA. Shown are results mean±SD from one donor of three independent donors used for this assay. **(B)** Lethal factor enzymatic activity is essential for IL-22 suppression in human tonsillar lymphocytes. Human tonsillar lymphocytes were treated with lethal toxin or E687C mutant lethal toxin (1.0 μg/ml) for 3 hr followed by IL-23 (50 ng/ml) stimulation for 18 hr. Cell supernatants were analyzed for IL-22 production by ELISA. Shown is mean±SD of one donor performed in triplicate from three independent donors.
(PDF)

S3 Fig Related to Fig 3 lethal toxin does not affect viability in MNK-3 cells. Lethal toxin did not cause apoptosis or necrosis in MNK-3 cells. MNK-3 cells were treated with lethal toxin (1.0 μg/ml) for 2 hr followed by IL-23 stimulation for 18 hr. Apoptosis was assessed by Annexin V and 7-AAD staining and flow cytometry. (**A**) Shown are representative plots from one experiment of two performed. Quantified apoptosis data and IL-22 secretion from the same experiment are shown in **B** and **C,** respectively. * p≤0.05, ** p≤0.01, *** p<0.001, **** p<0.0001 and non-significant (ns) p>0.05 by one-way ANOVA with Tukey’s post-hoc test.
(PDF)

S4 Fig Related to Fig 4 CD127+ ILCs expand in vitro to generate IL-22-producing ILC3s. **(A)**Gating strategy for sorting CD127^+^ ILCs. Tonsillar lymphocytes were depleted of CD19^+^ B cells using the eBioscience Magnisort CD19 positive selection kit. CD19 depleted-tonsillar lymphocytes were sorted for CD3^-^ CD19^-^ CD14^-^ CD56^-^ CD127^+^ ILCs. Cells were allowed to expand for at least 21 days in RPMI media supplemented with IL-2 (20 ng/ml), IL-7 (20 ng/ml), SCF (20 ng/ml), IL-15 (10 ng/ml) and FLT3L (10 ng/ml). **(B)** Surface characterization of *in vitro* expanded ILCs. *In vitro* expanded ILCs were stained with markers for CD3, CD19, CD14, CD127, c-Kit, CD161 and NKp44 and analyzed by flow cytometry. ILC3 were defined as CD3^-^ CD19^-^ CD14^-^ CD127^+^ c-kit^+^ CD161^+^. **(C)** IL-22 and GM-CSF production in *in vitro* expanded ILCs. *In vitro* expanded ILCs were stimulated with IL-1β, IL-23, PMA, ionomycin or a combination of these stimuli for 5 hr in presence of brefeldin A. Cells were analyzed by ICS and flow cytometry for IL-22 and GM-CSF.
(PDF)

S5 Fig Related to Fig 4 lethal toxin negatively modulates IL-1β-mediated IL-22 production by ILC3s. (**A**) MNK-3 cells were treated with or without 1 μg/ml lethal toxin (LeTx) or lethal factor only (LF) for 3 hrs and then stimulated with recombinant mouse IL-23 (50 ng/ml), IL-1β (20 ng/ml, from eBioscience) or no cytokine for 18 hrs. IL-22 was quantitated by ELISA. Bars represent mean±SD (n = 3). (**B**) MNK-3 cells were treated or not with lethal toxin for 3 hrs and then were simulated with no cytokine, IL-23 or IL-1β for 5 hrs in the presence of brefeldin A. Cells were then intracellularly cytokine stained for IL-22 and analyzed by flow cytometry. Number shown is the percent of cells within the gate. (**C**) MNK-3 cells were treated with no toxin or with lethal toxin (LeTx) for 3 hrs. Cells were then stimulated for 20 min with no cytokine (0), IL-1β or IL-23. Cell lysates were subjected to western blotting and sequentially probed with Abs to phosphorylated p38 (phospho-p38), total p38 or actin.
(PDF)

S6 Fig Related to Fig 6 gating strategy for identification of ILC3s from mice. (**A**) Shown is the gating strategy for identifying ILC3s from different tissues of lethal toxin treated or control mice. Cells were first gated for viability and then for lymphocyte size and granularity by forward and side scatter. CD45.2^+^ cells that were Lin (CD3, B220, CD11c, NK1.1)^-^ F4/80^-^ Thy1.2^+^ CD127^+^ were defined as ILC3s. (**B**) Shown is the gating strategy for identifying ILC3s that produce IL-22 and GM-CSF. After 5 hr stimulation with PMA, ionomycin and IL-23, cells were first gated for viability and then for lymphocyte size and granularity by forward and side scatter. CD45.2^+^ cells that were Lin (CD3, B220, CD11c, NK1.1, GR1)^-^ Thy1.2^+^ were examined for IL-22 and GM-CSF production.
(PDF)
