# Supplementary figures and images for: Bacillus anthracis lethal toxin negatively modulates ILC3 function through perturbation of IL-23-mediated MAPK signaling
Source: PLoS Pathog. 2017 Oct 23;13(10):e1006690. doi: 10.1371/journal.ppat.1006690 (PMC5695638; doi:10.1371/journal.ppat.1006690)

# Supplemental Figure 1

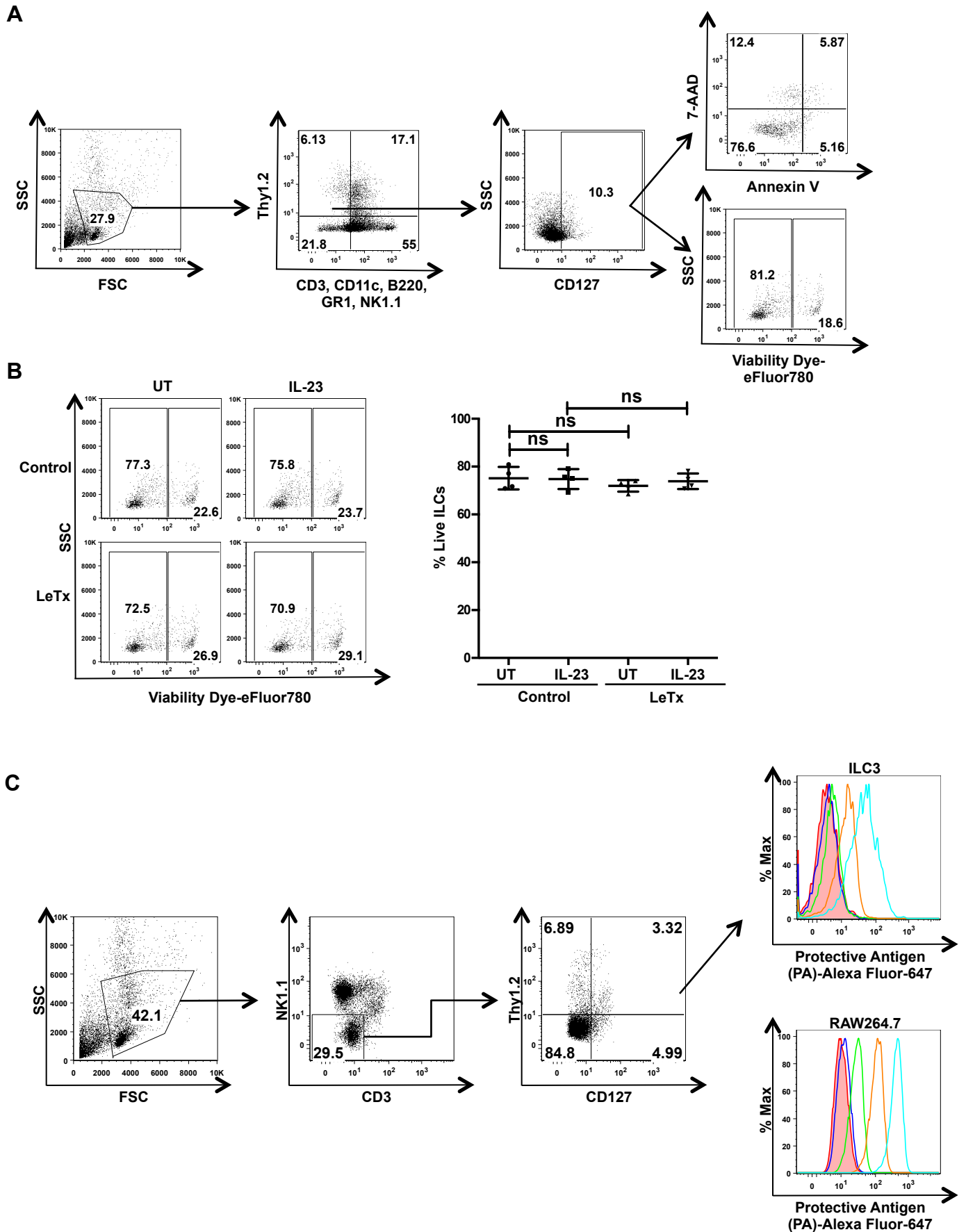

Supplement: S1 Fig — (A) Gating strategy for the apoptosis assay is shown. Cells were gated on forward and side scatter followed by Lin (CD3, CD11c, B220, GR-1 and NK1.1)- and Thy1.2+ CD127+ cells were analyzed for apoptosis by Annexin V and 7-AAD staining or for necrosis using viability eFluor780 dye. (B) Shown is a representative plot of viability eFluor780 stained samples (left) and the percentage of live cells (mean±SD, right) of a single representative experiment, shown in Fig 1D of 3 independent experiments. (C) Dose-dependent binding of mouse ILC3s (top) and RAW264.7, a macrophage cell line (bottom), and gating strategy for ILC3 of a representative experiment of 3 experiments is shown. Protective antigen (PA)-Alexa647 at indicated concentrations: 0 ug/ml (red), 0.01 μg/ml (blue), 0.1 μg/ml (green), 1 μg/ml (orange) and 10 μg/ml (cyan) were used to determine the binding to ILC3 or RAW264.7 mouse macrophages. (PDF) [file ppat.1006690.s001.pdf]

## Supplemental Figure 2

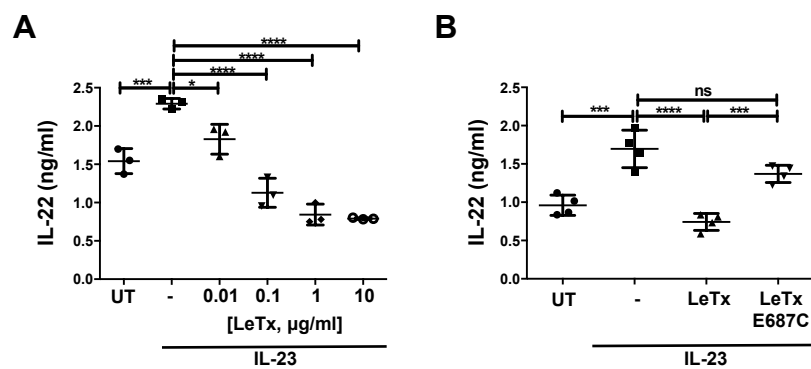

Supplement: S2 Fig — (A) Lethal toxin decreased IL-22 production in a dose-dependent manner in human tonsillar lymphocytes. Human tonsillar lymphocytes were treated with increasing concentrations (0.01–10 μg/ml) lethal toxin for 3 hrs followed by IL-23 (50 ng/ml) stimulation for 18 hr. Cell supernatants were analyzed for IL-22 secretion by ELISA. Shown are results mean±SD from one donor of three independent donors used for this assay. (B) Lethal factor enzymatic activity is essential for IL-22 suppression in human tonsillar lymphocytes. Human tonsillar lymphocytes were treated with lethal toxin or E687C mutant lethal toxin (1.0 μg/ml) for 3 hr followed by IL-23 (50 ng/ml) stimulation for 18 hr. Cell supernatants were analyzed for IL-22 production by ELISA. Shown is mean±SD of one donor performed in triplicate from three independent donors. (PDF) [file ppat.1006690.s002.pdf]

Supplemental Figure 3

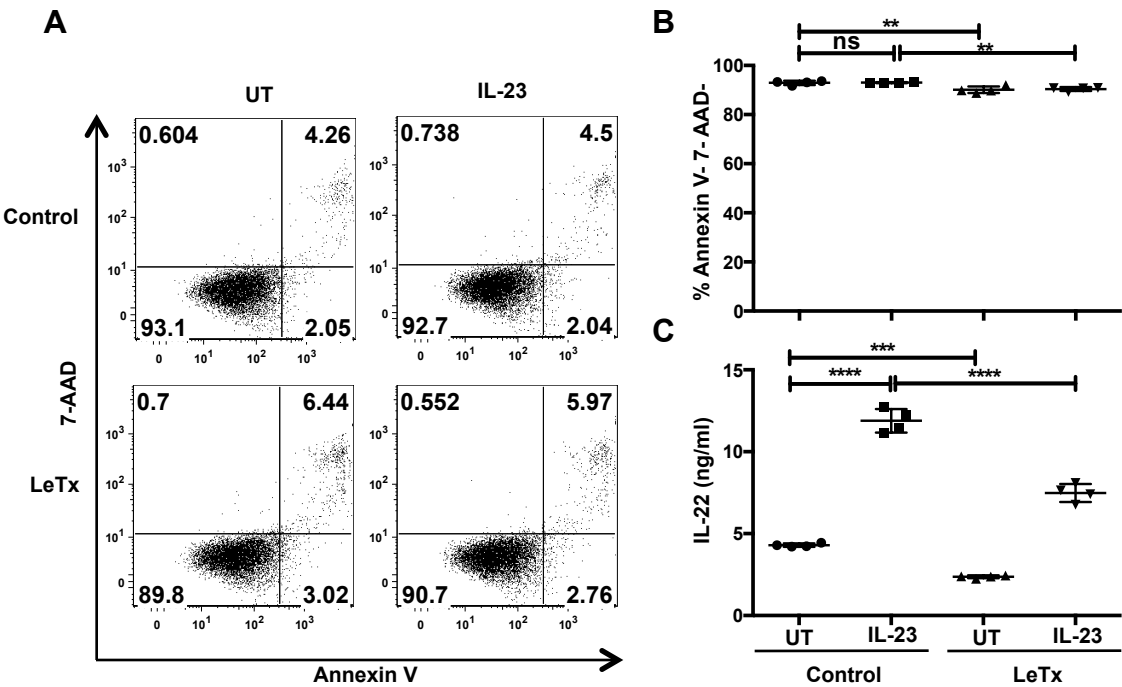

Supplement: S3 Fig — Lethal toxin did not cause apoptosis or necrosis in MNK-3 cells. MNK-3 cells were treated with lethal toxin (1.0 μg/ml) for 2 hr followed by IL-23 stimulation for 18 hr. Apoptosis was assessed by Annexin V and 7-AAD staining and flow cytometry. (A) Shown are representative plots from one experiment of two performed. Quantified apoptosis data and IL-22 secretion from the same experiment are shown in B and C, respectively. * p≤0.05, ** p≤0.01, *** p<0.001, **** p<0.0001 and non-significant (ns) p>0.05 by one-way ANOVA with Tukey’s post-hoc test. (PDF) [file ppat.1006690.s003.pdf]

# Supplemental Figure 4

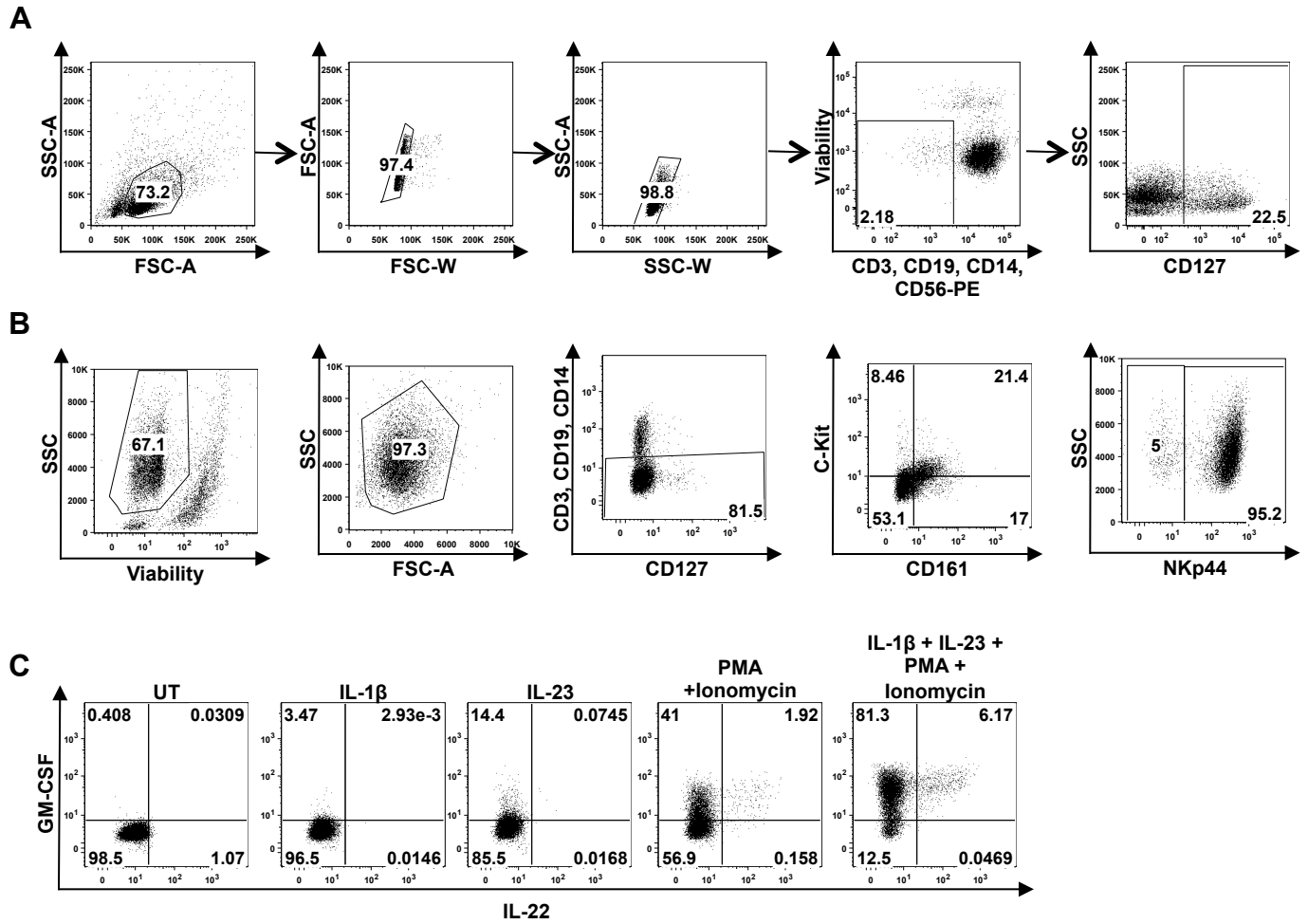

Supplement: S4 Fig — (A)Gating strategy for sorting CD127+ ILCs. Tonsillar lymphocytes were depleted of CD19+ B cells using the eBioscience Magnisort CD19 positive selection kit. CD19 depleted-tonsillar lymphocytes were sorted for CD3- CD19- CD14- CD56- CD127+ ILCs. Cells were allowed to expand for at least 21 days in RPMI media supplemented with IL-2 (20 ng/ml), IL-7 (20 ng/ml), SCF (20 ng/ml), IL-15 (10 ng/ml) and FLT3L (10 ng/ml). (B) Surface characterization of in vitro expanded ILCs. In vitro expanded ILCs were stained with markers for CD3, CD19, CD14, CD127, c-Kit, CD161 and NKp44 and analyzed by flow cytometry. ILC3 were defined as CD3- CD19- CD14- CD127+ c-kit+ CD161+. (C) IL-22 and GM-CSF production in in vitro expanded ILCs. In vitro expanded ILCs were stimulated with IL-1β, IL-23, PMA, ionomycin or a combination of these stimuli for 5 hr in presence of brefeldin A. Cells were analyzed by ICS and flow cytometry for IL-22 and GM-CSF. (PDF) [file ppat.1006690.s004.pdf]

Supplemental Figure 5

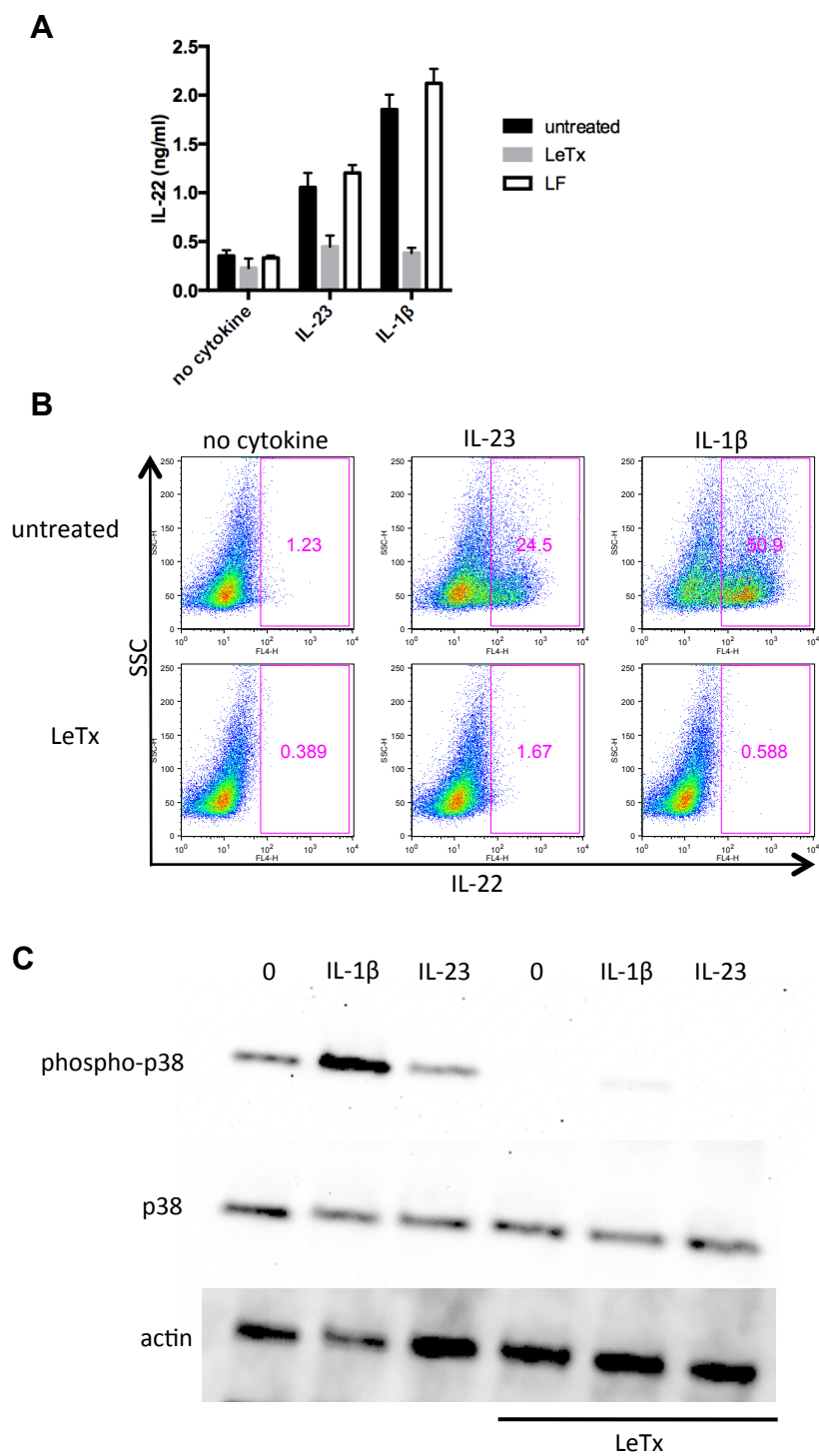

Supplement: S5 Fig — (A) MNK-3 cells were treated with or without 1 μg/ml lethal toxin (LeTx) or lethal factor only (LF) for 3 hrs and then stimulated with recombinant mouse IL-23 (50 ng/ml), IL-1β (20 ng/ml, from eBioscience) or no cytokine for 18 hrs. IL-22 was quantitated by ELISA. Bars represent mean±SD (n = 3). (B) MNK-3 cells were treated or not with lethal toxin for 3 hrs and then were simulated with no cytokine, IL-23 or IL-1β for 5 hrs in the presence of brefeldin A. Cells were then intracellularly cytokine stained for IL-22 and analyzed by flow cytometry. Number shown is the percent of cells within the gate. (C) MNK-3 cells were treated with no toxin or with lethal toxin (LeTx) for 3 hrs. Cells were then stimulated for 20 min with no cytokine (0), IL-1β or IL-23. Cell lysates were subjected to western blotting and sequentially probed with Abs to phosphorylated p38 (phospho-p38), total p38 or actin. (PDF) [file ppat.1006690.s005.pdf]

Supplemental Figure 6

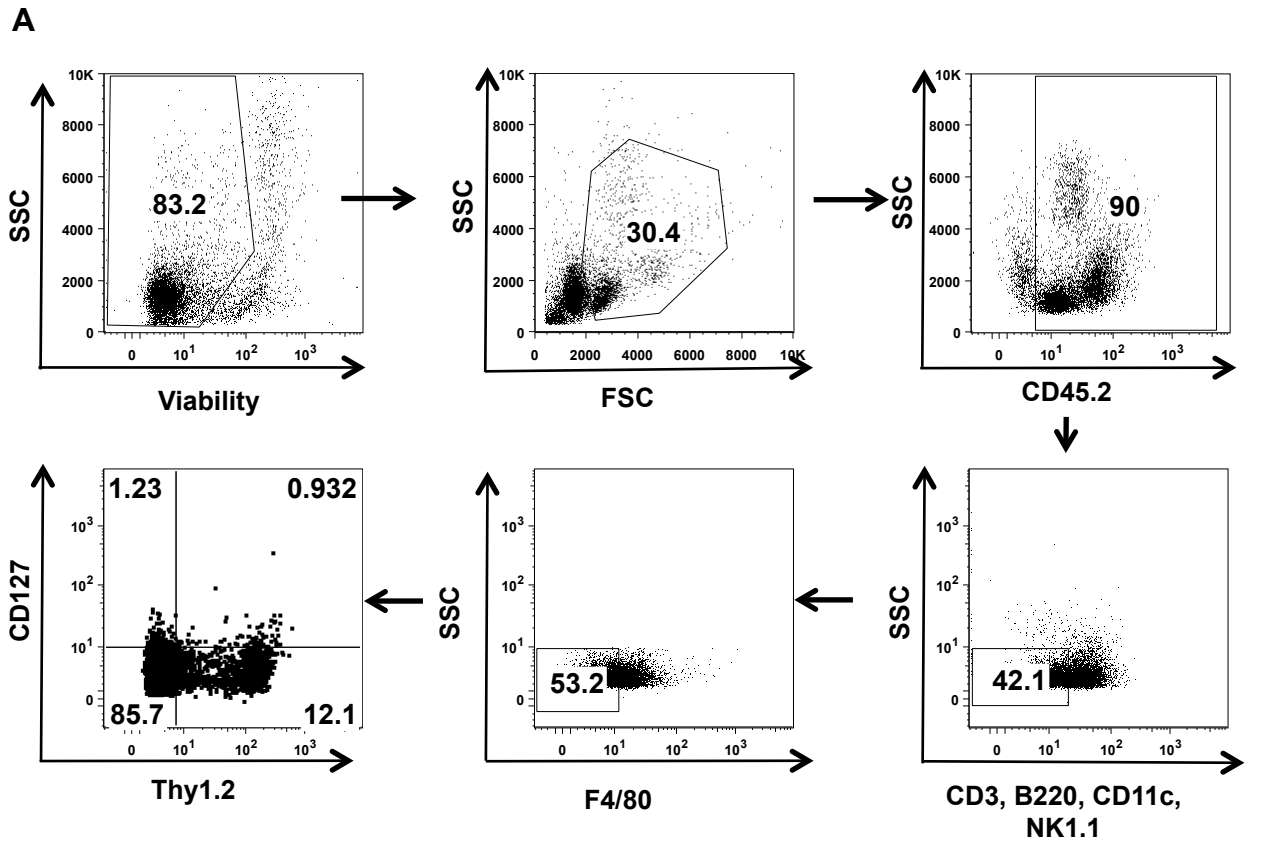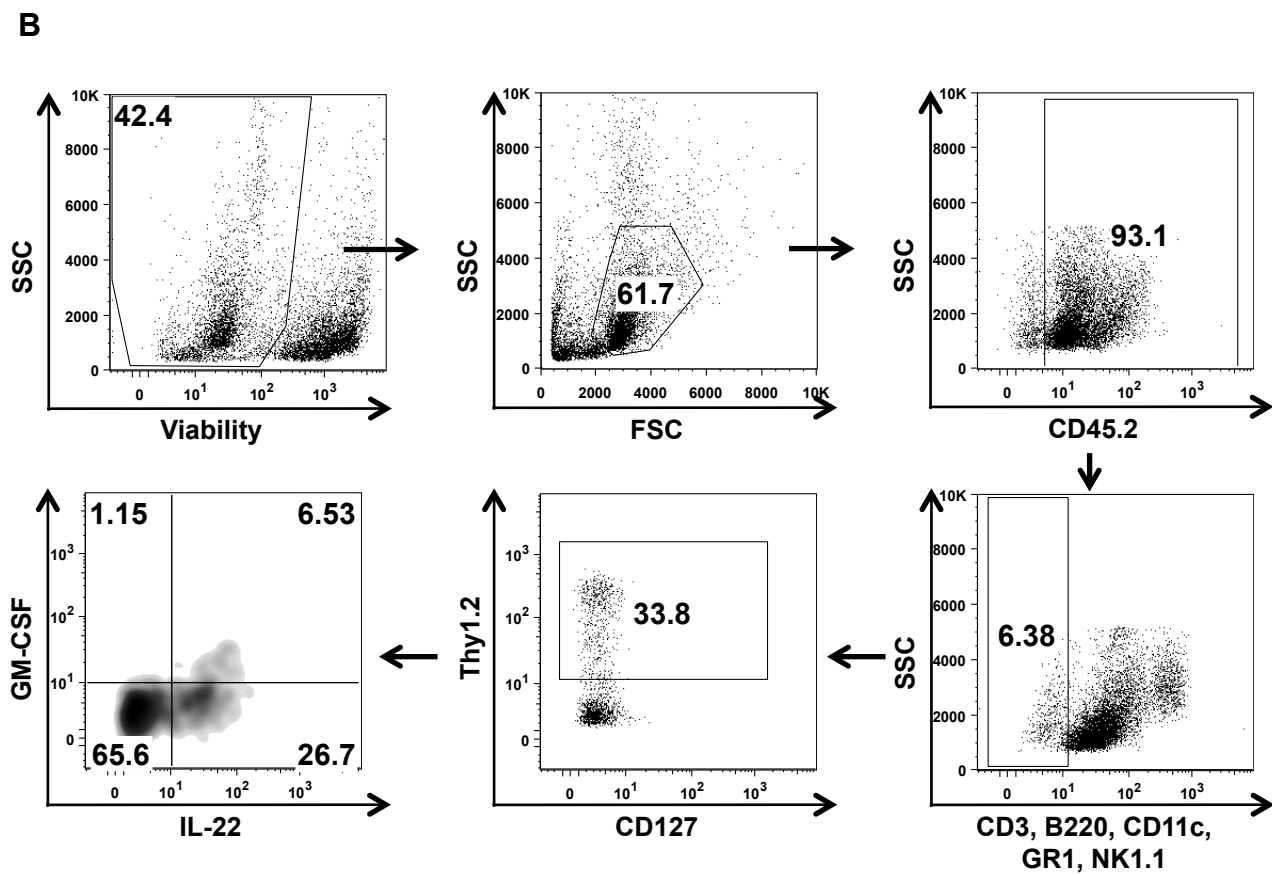

Supplement: S6 Fig — (A) Shown is the gating strategy for identifying ILC3s from different tissues of lethal toxin treated or control mice. Cells were first gated for viability and then for lymphocyte size and granularity by forward and side scatter. CD45.2+ cells that were Lin (CD3, B220, CD11c, NK1.1)- F4/80- Thy1.2+ CD127+ were defined as ILC3s. (B) Shown is the gating strategy for identifying ILC3s that produce IL-22 and GM-CSF. After 5 hr stimulation with PMA, ionomycin and IL-23, cells were first gated for viability and then for lymphocyte size and granularity by forward and side scatter. CD45.2+ cells that were Lin (CD3, B220, CD11c, NK1.1, GR1)- Thy1.2+ were examined for IL-22 and GM-CSF production. (PDF) [file ppat.1006690.s006.pdf]
